# The lived experience of patients with feet affected by RA: a thematic synthesis of the qualitative literature

**DOI:** 10.1186/1757-1146-8-S2-O38

**Published:** 2015-09-22

**Authors:** Matthew Love, Steven Walmsley, Stefania Penkala

**Affiliations:** 1 Department of Podiatric Medicine, School of Science and Health, University of Western Sydney, Sydney, Campbelltown, 2751, NSW, Australia

**Keywords:** Rheumatoid arthritis, lived experience, thematic synthesis, foot

## Background

Foot involvement in rheumatoid arthritis has detrimental and far reaching implications for the lives of patients. Qualitative studies investigating this phenomenon have identified specific aspects of the lived experience pertaining to the foot. However, no studies have attempted to provide a holistic overview of this phenomenon or investigate the potential relationships between different facets of the lived experience. The aim of this research was to synthesise the existing qualitative research and develop a model to conceptualise the patient lived experience.

## Methods

A systematic review and thematic synthesis of qualitative studies investigating the lived experience of patients with RA with reference to or focus on foot involvement was conducted. Electronic databases were searched (Embase, PsychINFO, MEDLINE and CINAHL), with no restriction on year of publication, along with the reference lists of relevant articles. The thematic synthesis was performed using qualitative data analysis software: EPPI-Reviewer 4.

## Results

Nineteen studies were included in the study, representing a collective total of 393 patients. A total of 7 major interactive themes, representative of the holistic lived experience were identified: arising psychological impacts (body image, negative feelings, perceptions of others), experiences with footwear (prescription footwear, choosing and changing shoes, wearing insoles), the healthcare journey (actively seeking/not seeking foot care, negotiating care pathways, foot healthcare provision), day-to-day living (family, activities of daily living, recreation and socialising, career/occupation), manifestations of RA in the foot (pain phenomena, swelling/inflammation, stiffness), bodily impacts of RA (bodily impacts, bodily appearance) and ways of coping (footwear strategies, reliance on others, stoicism, concealment, healthcare system, adaptions and compromise).

## Conclusions

When considered in a holistic context, RA involvement in the foot has diverse, interactive and substantial consequences for the daily lives of patients. A multitude of experiences ranging from navigating the healthcare system and quality of care provided to body image, footwear experiences, and family life are balanced against an array of different coping strategies. The findings from this study will offer health professionals insight and understanding into the holistic patient experience of RA involvement in the foot and potentially inform clinical practice, helping to optimise treatment and quality of life outcomes.

